# Conditional survival and annual hazard of death in older patients with esophageal cancer receiving definitive chemoradiotherapy

**DOI:** 10.1186/s12877-024-04939-w

**Published:** 2024-04-17

**Authors:** Xiao Chang, Wei Deng, Rong Yu, Weihu Wang

**Affiliations:** Key Laboratory of Carcinogenesis and Translational Research (Ministry of Education/Beijing), Department of Radiation Oncology, Peking University Cancer Hospital and Institute, 100142 Beijing, China

**Keywords:** Esophageal cancer, Older, Conditional survival, Death hazard

## Abstract

**Background:**

Definitive chemoradiotherapy is one of the primary treatment modalities for older patients with esophageal cancer (EC). However, the evolution of prognosis over time and the factors affected non-EC deaths remain inadequately studied. We examined the conditional survival and annual hazard of death in older patients with EC after chemoradiotherapy.

**Methods:**

We collected data from patients aged 65 or older with EC registered in the Surveillance, Epidemiology, and End Results database during 2000–2019. Conditional survival was defined as the probability of survival given a specific time survived. Annual hazard of death was defined the yearly event rate. Restricted cubic spline (RCS) analysis identified the association of age at diagnosis with mortality.

**Results:**

Among 3739 patients, the 3-year conditional overall survival increased annually by 7-10%. Non-EC causes accounted for 18.8% of deaths, predominantly due to cardio-cerebrovascular diseases. The hazard of death decreased from 40 to 10% in the first 6 years and then gradually increased to 20% in the tenth year. Non-EC causes surpassed EC causes in hazard starting 5 years post-treatment. RCS indicated a consistent increase in death hazard with advancing age, following a linear relationship. The overall cohort was divided into two groups: 65–74 and ≥ 75 years old, with the ≥ 75-year-old group showing poorer survival and earlier onset of non-EC deaths (HR = 1.36, 95% CI: 1.15–1.62, *P* < 0.001). Patients with early-stage disease (I-II) had higher risks of death from non-EC causes (HR = 0.82, 95% CI: 0.68–0.98, *P* = 0.035). Tumor histology had no significant impact on non-EC death risk (HR = 1.17, 95% CI: 0.98–1.39, *P* = 0.081).

**Conclusions:**

Survival probability increases with time for older patients with EC treated with chemoradiotherapy. Clinicians and patients should prioritize managing and preventing age-related comorbidities, especially in older cohorts and those with early-stage disease.

## Background

As global populations continue to age, the incidence of cancer is expected to rise significantly. By the year 2030, it is projected that approximately 70% of new cancer cases diagnosed will be in the older [1–3]. Among the various types of cancer, esophageal cancer (EC) is particularly common in older individuals, with more than 60% of new cases being diagnosed in patients aged 65 years or older. Definitive chemoradiotherapy (CRT) is currently the standard treatment modality for inoperable locoregional EC, particularly among the older population [4–7]. Unfortunately, the prognosis remains poor, with the 5-year overall survival (OS) rate being only 21.7% [8]. Uncertainty about prognosis can cause psychological stress and even affect post-treatment prognosis [9, 10]. 

Conditional survival is an important concept in the field of oncology [11–15]; it refers to the dynamic change in the likelihood of survival over time after diagnosis or treatment [16, 17]. This dynamic measurement can provide updated survival estimates at specific time points after treatment and help guide long-term follow-up strategies. Older patients with EC often face complex health challenges due to the presence of multiple comorbidities [18], but conditional survival and changes in cause of death over time have not been well studied in this age group.

This study aimed to assess the conditional survival probability and change in annual hazard of death over time in patients diagnosed with EC at the age of 65 years or older and treated with definitive CRT. The study findings could help clinicians better understand the disease process and improve overall management.

## Methods

### Patients selection

**Fig. 1:**
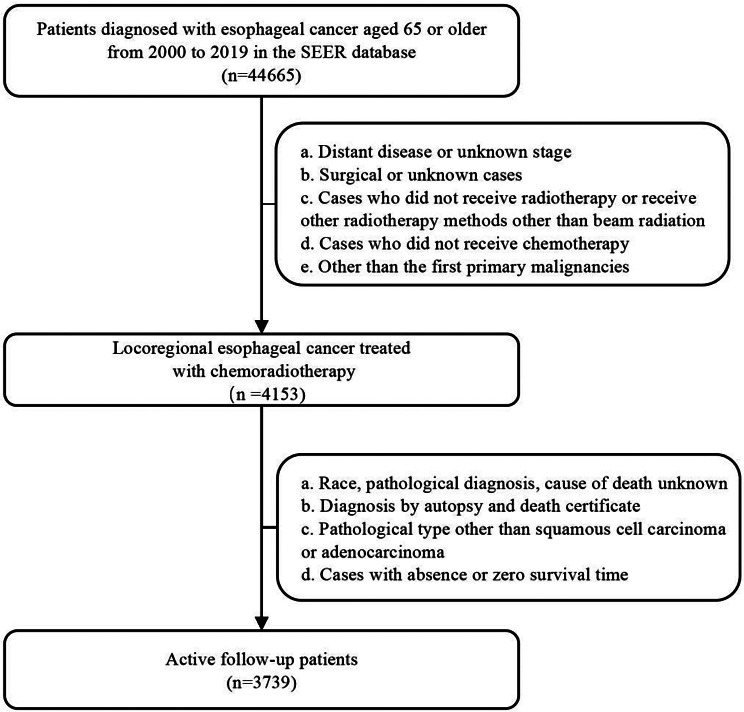
Diagram showing study sample selection process. SEER, Surveillance, Epidemiology, and End Results

The Surveillance, Epidemiology, and End Results (SEER) database was searched to identify patients diagnosed with EC from 2000 to 2019. Permission was obtained (reference number 11,564-Nov2019) to access SEER Research Plus Data, 17 Registries, Nov 2021Sub (2000–2019). Patients were eligible for inclusion in this study if they (1) were ≥ 65 years old; (2) had pathologically confirmed EC diagnosed as locoregional disease according to SEER criteria. (3) had received definitive CRT; and (4) EC served as the first primary malignancies. We excluded patients with (1) unknown age or missing data on summary stage or survival time; and (2) diagnosis established solely through autopsy or death certificate (Fig. 1).

This study was granted an exemption from review by the institutional review board as only de-identified preexisting data were used.

### Definitions

EC was classified as per the International Classification of Diseases for Oncology (ICD-0-3) codes and included adenocarcinoma (8140–8389, 8480, 8481, 8570, 8574), and squamous cell carcinoma (8050–8089). The location of the primary tumor was classified as thoracic upper (C15.0 and C15.3), middle (C15.4 and C15.1), and lower (C15.2 and C15.5). During the data cleaning process, the TNM stage was reclassified as accurately as possible in accordance with the AJCC 8th edition staging system.

### Statistical analysis

Statistical analysis was conducted using R 4.1.0 (https://www.r-project.org/). OS was defined as the time from the start of treatment to death from any cause. Cancer-specific survival (CSS) was defined as the time from the start of treatment to death from EC. Other cause–specific survival (OCSS) was defined as the time from the start of treatment to death from non-EC causes (e.g., cardiovascular disease). Considering the unique circumstances of older patients, we chose 3 years as the time horizon for cancer care instead of the conventional 5 years. Conditional OS was introduced to assess the probability of surviving for an additional number of years, given that a patient had already survived a certain number of years from the start of treatment [16]. Thus, the 3-year conditional OS at 2 years was the conditional probability of surviving an additional 3 years, given that the patient had already survived 2 years. Additionally, extensions of the concept of conditional survival were applied to determine conditional CSS and OCSS, i.e., the probability of CSS or OCSS for an additional number of years given that the patient had survived a certain number of years without death from EC or other causes. The survival rate was estimated by the Kaplan–Meier method and compared using the log-rank test. Conditional survival was calculated by using the “condsurv” package in R [19]. 

Annual hazard of death was estimated as the annual number of deaths divided by the number of patients at risk in that year and was depicted with smoothed hazard curves. The annual hazard rate was calculated for the overall cohort and subgroups of cancer histology, age, and cancer stage. The restricted cubic spline (RCS) analysis was used to identify the association of age at diagnosis with mortality, adjusted for histology, stage, grade, race, and other relevant factors. All tests were two-sided, and the statistical significance was at *P* < 0.05.

## Results

### Baseline characteristics

Table 1 presents the baseline characteristics of the 3739 patients included in this study. The median age of the patients was 74 years, and the male-to-female ratio was 3:1. While 2072/3739 (55.4%) patients had adenocarcinoma, 1667/3739 (44.6%) had squamous cell carcinoma. Most than half of the lesions (56.3%) had spread beyond the esophageal wall (T3-4), and 56.2% of the lesions were located in the lower esophagus. A large proportion of patients (42.3%) presented with node-positive disease, and approximately 75% of patients had stage II-III disease. After a median follow-up of 63 months, the 1-, 3- and 5-year OS rates were 58%, 26.8%, and 16.6%, respectively, with a median OS of 16 months. The 1-, 3- and 5-year CSS rates were 62.5%, 33.0%, and 24.3%, respectively, with a median CSS of 18 months. In the overall cohort, 57.5% (2151/3739) died from EC, while 18.8% (702/3739) died from non-EC causes. Cardiovascular and cerebrovascular diseases were the predominant causes of non-EC deaths, accounting for 198 (28.2%) deaths. Respiratory diseases (chronic obstructive pulmonary disease, pneumonia, and so on) followed closely behind, accounting for 102 (14.5%) deaths. Other causes of non-EC deaths included digestive tract disease (*n* = 92), neoplasm-related death (*n* = 59), and septicemia (*n* = 27).

**Table 1:** Clinical baseline characteristics of patients and tumors

Characteristics	Overall (*n* = 3739)	Characteristics	Overall (*n* = 3739)
Sex, n (%)		T-stage, n (%)	
Female	969 (25.9)	T1	808 (21.6)
Male	2770 (74.1)	T2	639 (17.1)
Age, years, median (IQR)	74.0 (69.0, 80.00)	T3	1823 (48.8)
Marital status, n (%)		T4	282 (7.5)
Married	2115 (56.6)	TX	187 (5.0)
Unmarried	1624 (43.4)	N-stage, n (%)	
Race, n (%)		N0	1326 (35.5)
Black	334 (8.9)	N1	1075 (28.8)
Other	239 (6.4)	N2	201 (5.4)
White	3166 (84.7)	N3	38 (1.0)
Histology, n (%)		N+	267 (7.1)
Squamous cell carcinoma	1667 (44.6)	Nx	832 (22.3)
Adenocarcinoma	2072 (55.4)	Stage, n (%)	
Location, n (%)		I	672 (18.0)
Upper	426 (11.4)	II	1314 (35.1)
Middle	889 (23.8)	III	1494 (40.0)
Lower	2101 (56.2)	IVA	15 (0.4)
Other	323 (8.6)	Other	244 (6.5)
Grade, n (%)			
I-II	1617 (43.2)		
III-IV	1420 (38.0)		
Unknown	702 (18.8)		

### Conditional survival and annual hazards over time for the overall cohort

**Fig. 2:**
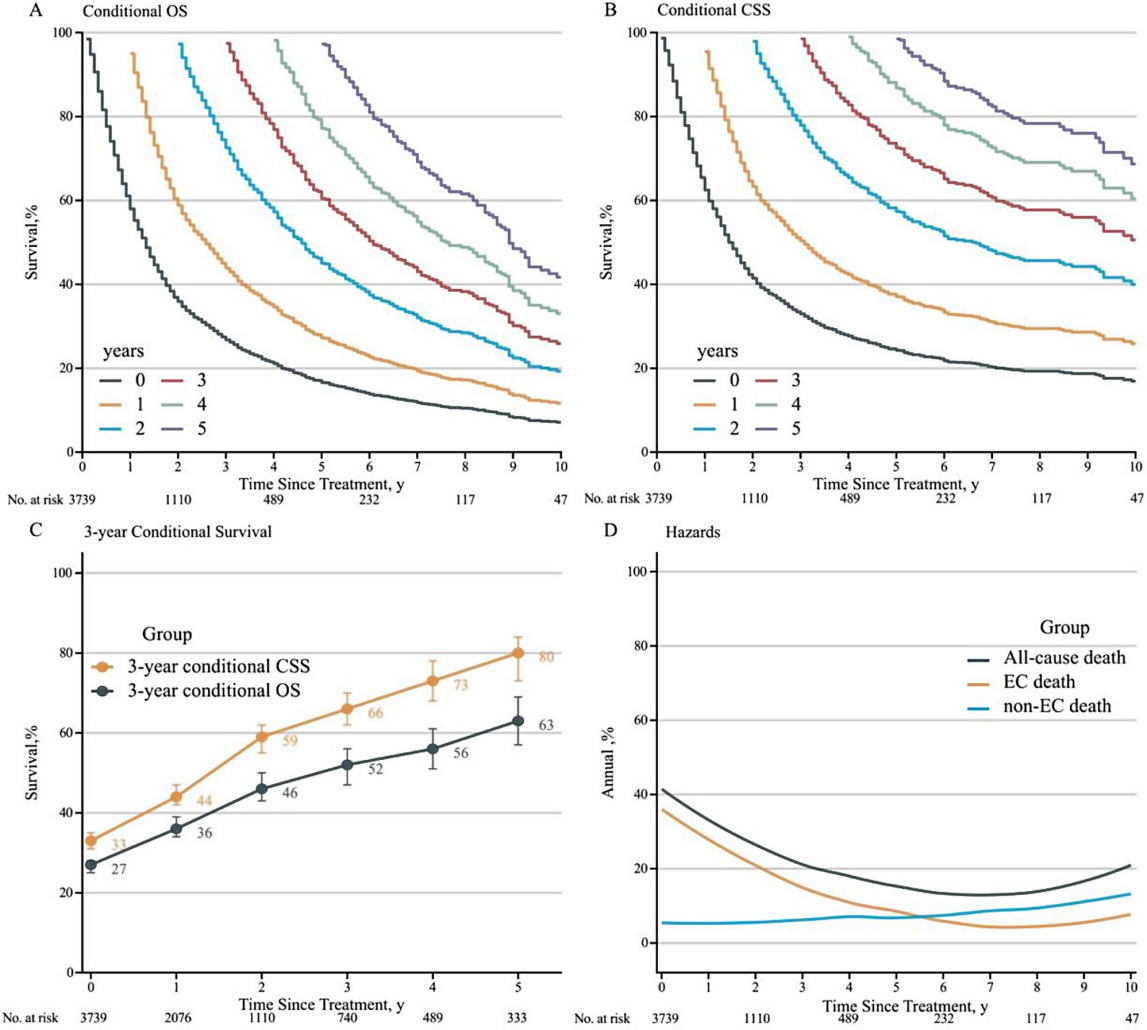
(**A**) Conditional overall survival (OS) curves as a function of the number of years survived since treatment. (**B**) Conditional cancer-specific survival (CSS) curves as a function of the number of years of cancer-specific survival since treatment. The colors of the lines are assigned in the order of years survived or cancer-specific survival since treatment, from years 0 to 5. (**C**) Three-year conditional survival probability as a function of the number of years survived or cancer-specific survival since treatment. (**D**) Smoothed hazard plots for annual rates of death from all causes, death from EC, and death from non-EC causes by time after treatment. OS, overall survival; CSS, cancer-specific survival; EC, esophageal cancer

Figure 2A and B show the survival curves for conditional OS and conditional CSS, respectively. Unlike the 3-year OS rate, the 3-year conditional OS rates exhibited a progressive increase, reaching 36% (95% CI, 34-39%) in patients with 1-year survivorship, 46% (95% CI, 43-50%) in those with 2-year survivorship, 52% (95% CI, 47-56%) in those with 3-year survivorship, 56% (95% CI, 51-61%) in those with 4-year survivorship, and 63% (95% CI, 57-69%) in those with 5-year survivorship. Likewise, the 3-year conditional CSS rates increased to 44% (95% CI, 42-47%) for patients with 1-year survivorship, 59% (95% CI, 55-62%) for those with 2-year survivorship, 66% (95% CI, 62-70%) for 3-year survivorship, 73% (95% CI, 68-78%) for 4-year survivorship, and 80% (95% CI, 73-84%) for 5-year survivorship. Notably, the 3-year conditional OS and CSS showed an initial rapid increase of more than 10% during the initial two years, with a less rapid increase thereafter (Fig. 2C). Figure 2D shows the dynamics of the annual hazard of all-cause death, death from EC, and death from non-EC causes. The death hazard from all causes exhibited a “U” shape, with the hazard rate decreasing from 40 to 10% in the first 6 years and then increasing to 20% in the tenth year. Additionally, for the overall cohort, the hazard of death from non-EC causes surpassed death from EC causes, starting 5 years after treatment.

### Annual hazard analysis in subgroups

**Fig. 3:**
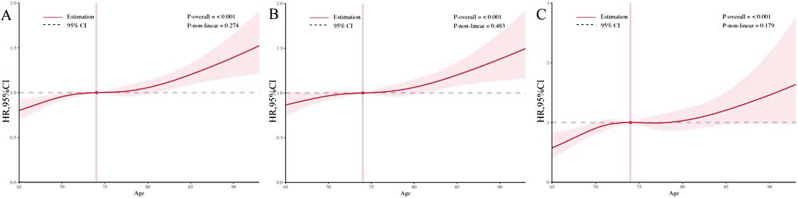
The restricted cubic spline plot for the association of age at diagnosis and hazard of death from all causes (**A**), EC cause (**B**), and non-EC causes (**C**). EC, esophageal cancer

The RCS plot (Fig. 3A-C) illustrates the relationship between age at diagnosis and hazard of all-cause death, death from EC, and death from non-EC causes. The hazard of death consistently increased with advancing age, following a linear relationship (all P-overall < 0.001 and P-non-linear > 0.05).

**Fig. 4:**
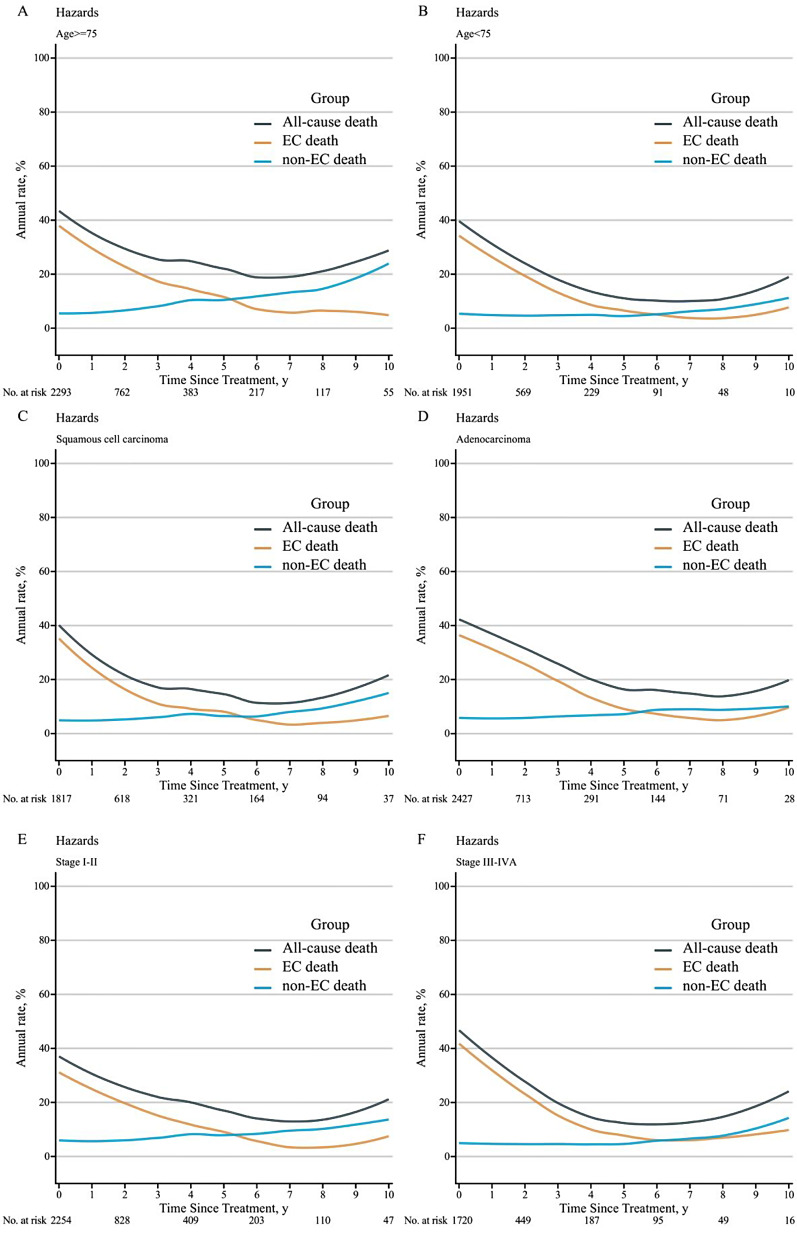
Smoothed hazard plots for annual rate of death from all causes, death from EC, and death from non-EC causes by time after treatment, stratified by the age (**A**- **B**), histology (**C**- **D**), and stage (**E**- **F**). EC, esophageal cancer

Using the median age of 74 years as the cutoff, the overall cohort was divided into two groups: a 65-74-year age group (*n* = 1964) and a ≥ 75-year age group (*n* = 1775). The ≥ 75-year age group had significantly inferior 3-year OS (23.9% vs. 29.4%, *P* < 0.001), and a higher risk of death from non-EC causes (HR = 1.36, 95% CI: 1.15–1.62, *P* < 0.001). Furthermore, the contribution of non-EC causes to death began earlier in the ≥ 75-year age group, starting at the fifth year versus the seventh year in the 65-74-year age group (Fig. 4A-B).

The 3-year OS rate was significantly higher in patients with squamous cell cancer than in patients with adenocarcinoma (32.2% vs. 22.2%, *P* < 0.001). The hazard of death from non-EC causes was similar in patients with the two cancer histologies (HR = 1.17, 95% CI: 0.98–1.39, *P* = 0.081). Additionally, non-EC deaths exceeded EC deaths by the fifth year for both cancer histologies (Fig. 4C-D).

Patients in stage I-II (*n* = 1986) showed significantly better survival than patients in stage III-IV (*n* = 1509), with 3-year OS rates of 31.0% vs. 21.6%, respectively (*P* < 0.001). Consequently, patients in stage I-II were more likely to die from non-EC causes (HR = 0.82, 95% CI: 0.68–0.98, *P* = 0.035). Moreover, the hazard of death from non-EC causes exceeded death from EC starting from the fifth year for patients in stage I-II compared to the seventh year for patients in stage III-IV (Fig. 4E-F).

## Discussion

In this study of 3739 older patients (aged 65 years or older) with EC treated with definitive CRT, we found that non-EC-related deaths, primarily deaths due to cardio-cerebrovascular and pulmonary diseases, accounted for approximately 25% of all deaths. Conditional OS and CSS consistently increased over time, with the annual hazard of death from non-EC causes surpassed that of death from EC starting from the fifth year post-treatment. The hazard of death demonstrated a linear increase with advancing age. Moreover, the risk of death from non-EC causes was significantly higher in older patients (≥ 75 years) and in those with early-stage (I-II) disease.

Non-cancer-related mortality poses a significant concern among cancer patients. Our study revealed that non-EC deaths comprised approximately 25% of all deaths, particularly attributed to cardio-cerebrovascular and pulmonary diseases. This heightened incidence of non-cancer-related mortality could potentially be associated with advanced age and adverse effects following radiation therapy. Studies have shown that high-dose radiation to the pericardium in inoperable patients with EC significantly increased the risk of pericardial effusion, while breast cancer radiotherapy had been linked to increased heart toxicity, especially in women with pre-existing ischaemic heart disease [3, 20]. These suggested that alongside tumor treatment, controlling treatment-related toxicity was imperative, as it can impact patients’ long-term survival.

Several studies have investigated the dynamic risk of cancer mortality [21, 22]. Our study observed a 7-10% annual increase in the estimated 3-year conditional overall survival rates, reaching 60% for patients surviving 5 years. The annual hazard of all-cause death exhibited a U-shaped pattern, suggesting a transition in causes of death over time. Initially, EC accounted for the majority of deaths, peaking at 40% in the first-year post-treatment. However, EC deaths decreased to 10% by the sixth year, while non-EC deaths became predominant. Over time, non-EC deaths gradually rose, reaching 15% by the tenth year. These findings aligned with the study by Chesney et al. [23], which found that non-cancer deaths surpassed cancer deaths among older patients of all cancer types at five years. This underscores the importance of addressing age-related comorbidities, particularly five years after definitive chemoradiotherapy.

The varying dynamics of annual death hazard among subgroups offer valuable insights into initial treatment and surveillance strategies. We observed a linear increase in death hazard with age, indicating no significant cutoff change in mortality risk between ages 65 and 75. Notably, the annual death hazard in the fifth year from non-EC causes in the ≥ 75 years age group was three times higher than that in the 65–74 age group (11.0% vs. 3.5%, *P* < 0.001). This emphasized addressing comorbidities in older cohorts during initial cancer treatment. Additionally, definitive chemoradiation yielded better outcomes for the squamous cell subtype compared with adenocarcinoma (3-year OS, 32.2% vs. 22.2%, *P* < 0.001), suggesting the need for new strategies in treating older patients with esophageal adenocarcinoma, with immunotherapy potentially being a promising option [24]. Tumor histology was not a prognostic factor for non-EC death (HR = 1.17, 95% CI: 0.98–1.39, *P* = 0.081). Interestingly, patients in stage I-II faced a higher risk of non-EC death than patients in stage III-IVA (HR = 0.82, 95% CI: 0.68–0.98, *P* = 0.035), possibly due to longer survival duration leading to increased likelihood of encountering non-EC deaths. Therefore, the more attention should be paid to treatment-related toxicity for the earlier the stage of the disease.

This study has several limitations. First, the lack of detailed information on chemotherapy regimens, radiation doses, radiation technique and treatment durations prevented in-depth analyses of treatment effects. However, the effect of these factors has been examined in previous studies and likely does not impact the results of our study. Second, our study focused only on patients who received chemoradiotherapy; so it is not possible to generalize our results to those undergoing curative surgery. Finally, the inability to assess pre-existing comorbidities and their impact on cardiovascular disease–related mortality can be considered a limitation.

## Conclusions

Among older patients with EC, the conditional overall survival increases over time at a rate of 7-10% per year, reaching 60% in patients who have survived for 5 years. Furthermore, there is a gradual transition in the annual hazard of death due to EC and other causes. Particularly in older cohort (≥ 75-year-old) and those with early-stage disease (I-II), the effective management of comorbidities throughout treatment and surveillance is crucial for survival benefits. Future research should aim to elucidate the relationship between non-esophageal cancer mortality and treatment toxicities in this age group.

## Data Availability

The datasets generated during the current study are publicly available in the SEER database (https://seer.cancer.gov).

## Abbreviations

CRT: Chemoradiotherapy
CSS: Cancer-specific survival
EC: Esophageal cancer
OCSS: Other cause-specific survival
OS: Overall survival
RCS: Restricted cubic spline
SEER: Surveillance, Epidemiology, and End Results
